# ExtraTrain: a database of Extragenic regions and Transcriptional information in prokaryotic organisms

**DOI:** 10.1186/1471-2180-6-29

**Published:** 2006-03-15

**Authors:** Eduardo Pareja, Pablo Pareja-Tobes, Marina Manrique, Eduardo Pareja-Tobes, Javier Bonal, Raquel Tobes

**Affiliations:** 1 Bioinformatics Unit, Era7 Information Technologies SL, BIC Granada CEEI, Parque Tecnológico de Ciencias de la Salud – Armilla Granada 18100, Spain

## Abstract

**Background:**

Transcriptional regulation processes are the principal mechanisms of adaptation in prokaryotes. In these processes, the regulatory proteins and the regulatory DNA signals located in extragenic regions are the key elements involved. As all extragenic spaces are putative regulatory regions, ExtraTrain covers all extragenic regions of available genomes and regulatory proteins from bacteria and archaea included in the UniProt database.

**Description:**

ExtraTrain provides integrated and easily manageable information for 679816 extragenic regions and for the genes delimiting each of them. In addition ExtraTrain supplies a tool to explore extragenic regions, named Palinsight, oriented to detect and search palindromic patterns. This interactive visual tool is totally integrated in the database, allowing the search for regulatory signals in user defined sets of extragenic regions. The 26046 regulatory proteins included in ExtraTrain belong to the families AraC/XylS, ArsR, AsnC, Cold shock domain, CRP-FNR, DeoR, GntR, IclR, LacI, LuxR, LysR, MarR, MerR, NtrC/Fis, OmpR and TetR. The database follows the InterPro criteria to define these families. The information about regulators includes manually curated sets of references specifically associated to regulator entries. In order to achieve a sustainable and maintainable knowledge database ExtraTrain is a platform open to the contribution of knowledge by the scientific community providing a system for the incorporation of textual knowledge.

**Conclusion:**

ExtraTrain is a new database for exploring Extragenic regions and Transcriptional information in bacteria and archaea. ExtraTrain database is available at .

## Background

TRANSFAC database [1] compiles eukaryotic cis-acting regulatory DNA elements and trans-acting factors covering from yeast to humans. However, a database for bacteria and archaea with a similar global approach it is not available. We can find information dealing with prokaryotic transcriptional regulation in RegulonDB [2] but it is limited to the network of transcriptional regulation in Escherichia coli K-12. There are other family oriented approaches like AraC-XylS [3] and BacTregulators [4] covering all bacteria and archaea but their knowledge contents are limited to two families.

Eukaryotic transcription factors usually bind a sufficiently numerous set of binding sites in a genome, allowing the determination of a motif for the DNA binding site for every transcription factor. Some comprehensive tools as PromoterPlot [5], MatInspector[6], TOUCAN [7], EZRetrieve [8], P-Match [9] or BEARR [10] are specifically oriented to the extraction and analysis of regulatory regions of mammalian genes. In contrast, in prokaryotes the majority of the regulators are very specific and usually have either just one DNA binding site or a very limited number of them in each genome and hence, it is not possible the definition of a DNA binding motif using data from only one genome. However, the increasing amount of available genomes of bacteria and archaea opens new possibilities for the definition of DNA binding motifs using the information about binding sites of orthologous proteins from different genomes. Comparative analysis of sequences of genes has been critical in the prediction of the function and structure of proteins, especially for developing the intensive task of annotation of genomes.

Moreover, there are interesting initiatives as coliBASE [11] oriented to comparative genomics but they are also centred in genes. However comparative analysis of extragenic regions from bacteria remains almost unexplored.

ExtraTrain follows an integrative approach with a special focus on DNA extragenic regions as the target of regulatory proteins, providing a new platform for analyzing transcriptional regulation in prokaryotes. ExtraTrain includes all extragenic regions corresponding to all completely annotated genomes of bacteria and archaea available at NCBI [12] and all regulatory proteins included in UniProt [13] belonging to all the most significant families of transcriptional regulatory proteins (excluding sigma factors) defined in prokaryotes.

In response to the need of integration of biological databases we have adopted the UniProt definition of entry, based solely on amino acid sequence. However, the function and regulation of a protein does not only depend on its sequence, but also on its genetic context. Thus, two genes encoding exactly the same protein but with different regulatory signals in their upstream regions, can play different functional roles in an organism. Moreover, two identical genes with identical upstream extragenic regions can play different roles if they belong to different organisms because the regulatory network for each of them can be different. In each ExtraTrain regulatory protein entry the different genetic contexts can be explored clicking on the extragenic regions listed in the section "UPSTREAM extragenic regions corresponding to this protein". This strategy allows us both to contemplate the genetic context and to maintain only one entry for each protein, preserving thus a complete integration with Uniprot.

## Construction and content

Programs in Java have been developed for the task of constructing and reconstructing the database with raw data from UniProt and NCBI genome database.

ExtraTrain runs on a server having Apache as web server, MySQL as database management system and Macromedia ColdFusion as Application Server.

The interactive tool to explore extragenic sequences (Palinsight) has been developed using Macromedia Flash.

The actualization and the maintenance of the automatically acquired data about extragenic regions and regulatory proteins are managed by releases. However, the reference database and the knowledge data contributed by researchers will be continuously updated.

ExtraTrain includes data in relation to:

### • Extragenic regions

All DNA extragenic regions and the information of the upstream and downstream genes of available genomes of bacteria and archaea are included in ExtraTrain. We have included not only the extragenic regions corresponding to regulatory proteins but all extragenic regions of each genome. Thus, each regulatory protein can be analyzed in its genetic context having available all its possible DNA targets. ExtraTrain includes data corresponding to the 230 genomes available at NCBI on 11 July 2005.

### • Regulatory proteins

The set of proteins is extracted from 10-5-2005 release of UNIPROT (SwissProt +TrEMBL) database. The 26046 proteins are classified in 16 families: AraC/XylS, ArsR, AsnC, Cold shock domain (CSD), CRP-FNR, DeoR, GntR, IclR, LacI, LuxR, LysR, MarR, MerR, NtrC/FIS, OmpR and TetR. We have followed the InterPro definition of each family. The entries of the InterPro database [14] used to define each family and the number of members of each family included in ExtraTrain are displayed in Table 1.

### • BLAST similarity

"All against all" BLAST analysis has been carried out within the members of each family of regulators. These results are stored in the database allowing fast access to similarity data. It also allows us to offer the possibility of selecting a set of extragenic regions upstream BLAST similar regulators (See case study below).

### • References

ExtraTrain includes a set of references extracted from Medline and manually curated by experts. These references are associated with specific protein entries of the database, with specific families or with other ExtraTrain items.

### • Textual knowledge

ExtraTrain offers a system for the incorporation of knowledge by scientists. Each knowledge unit is always associated to a Medline reference and can be associated to one of eight different fields: function, regulated genes, regulatory network, 3D-structure, mutations, DNA-binding, effectors and applications. Each input of knowledge is signed by the contributor.

We have connected the data of genes extracted from NCBI genome resource with the protein data from UniProt databases. Thus, each ExtraTrain extragenic region entry displays UniProt data for the two proteins encoded by the genes delimiting the extragenic space. For transcriptional regulatory proteins we have also established the connexion between each protein and all their available genetic contexts. It allows to obtain for each protein the different extragenic regions that have been found upstream its corresponding gene in all available genomes.

## Utility and discussion

The purpose of ExtraTrain is to provide a platform to easily manage extragenic regions and transcriptional regulators in bacteria and archaea.

### User interface

#### Extragenic region entry

In ExtraTrain "extragenic region" is defined as the DNA space between two genes of a genome. The extragenic region entry displays the sequences of the extragenic region and the proteins codified by the two bordering genes. The positive or negative orientation of each gene and their positions in the genome element are also indicated. Links to NCBI data about the two genes and the two entries of UniProt corresponding to the encoded proteins are also provided. Two arrows to navigate backward and forward along the chromosome are available to explore neighbouring genes and extragenic regions. Thus, the user can easily move along a genome element visualizing all their genes and extragenic regions. It facilitates the evaluation of the genetic context.

#### Extragenic region search tools

The extragenic region search page offers the possibility of selecting a specific genome extragenic region by introducing either its ExtraTrain ID or the RefSeq protein ID. For this last option the user can select either to obtain the upstream or the downstream extragenic region. ExtraTrain offers several options for the selection of a set of extragenic regions within a genome element:

a. extragenic regions upstream or downstream regulators of a specific family

b. extragenic regions included within a genome fragment defined by introducing an initial and a final position

c. selection of extragenic regions in which an exact pattern of sequence is present.

The user can easily 'shop' for extragenic sequences by querying the database using the described search tools. Sets of extragenic sequences selected by the different options can be combined in an up to 100 extragenic sequences common set that we have named "working set". The construction of this "working set" is easy and interactive facilitating the access and selection of extragenic sequences. We realized that the accessibility to sequences of genes and proteins was fast and easy through several resources while the access to extragenic sequences used to be more difficult. Many bench scientists without IT background can find difficulties in the access and management of prokaryotic extragenic sequences. One of the purposes of ExtraTrain is to provide a user-friendly platform for the management of these extragenic sequences. The user can obtain the extragenic sequences included in the "working set" in FASTA format as well as the inverted complementary sequence for each of them. This option is very useful to align extragenic sequences upstream orthologous genes that are allocated in different DNA strands. The "working set" in FASTA format can be used as input for external pattern discovery tools. We provide links to the web interfaces of the tools Bioprospector, AlignAce, ANN-Spec, Consensus, Improbizer, MEME, MITRA, MotifSampler, Oligo/dyad-analysis, QuickScore, SeSiMCMC and YMF whose limitations and potentials have been recently assessed [15,16]. The user can also send the "working set" to Palinsight (See below).

#### Transcriptional regulatory proteins

An initial page about regulatory proteins displays information about the 16 families included in the database (Table 1) and a graphic illustration representing the distribution of families in the ExtraTrain database. By selecting a genome the user can obtain a graphical view displaying the distribution of the different families of regulatory proteins in the selected genome.

The ExtraTrain definition of entry for transcriptional regulatory proteins is identical to the UniProt definition of entry, which is based solely on the protein sequence.

Furthermore, the ExtraTrain regulator entry identifier is the UniProt identifier for this protein. We have chosen these unified criteria to reinforce invaluable initiatives of integration such as UniProt.

The web page corresponding to an ExtraTrain regulatory protein entry shows data automatically extracted from Uniprot, manually curated references extracted from Medline, a list of BLAST similar proteins and information about all extragenic regions upstream this regulator in the available genomes. The majority of regulatory proteins in the current ExtraTrain database are encoded by only one gene in one genome and hence, have only one upstream extragenic region. However, in the near future, with the availability of several strains for each species, it will be frequent to found several genes in several genomes encoding the same regulatory protein. It will allow the analysis of each protein in its different genetic contexts. This lack of one to one relationship between proteins and genes is solved in ExtraTrain by establishing the connexion between proteins and genes through extragenic regions without the loss of biologically relevant information.

#### Regulatory protein search tools

The regulator search web page allows access to one specific regulator introducing either its UniProt identifier or its RefSeq protein ID. The selection of sets of regulators sharing an InterPro ID or a COG ID is also available. Another search option is the text search within a family. The text search can also be restricted to a specific genome.

#### Palinsight: visual tool for palindromic pattern detection

Identification of regulatory motifs is crucial in the study of gene expression.

Transcription factors are proteins that frequently adopt antiparallel dimeric structures that bind DNA palindromic motifs. These motifs are often highly divergent in sequence but in many cases share conserved palindromic motifs. Nowadays, there are available sophisticated tools for the multiple alignment of sequences that facilitate the discovery of sequence motifs. However, when working with DNA sequences there often arises the need to inspect DNA palindromy, even independently of sequence similarity. This palindromy inspection is especially required when dealing with non coding regulatory DNA sequences. To perform this task manually is very time consuming and error prone. We have incorporated within the ExtraTrain database a palindromy viewer and searcher to explore palindromy in DNA extragenic regions. Palinsight allows to analyze up to 100 sequences distributed in screens displaying 10 sequences. The tool allows the visualization of the palindromy of each sequence in each of the possible palindromy axes in an interactive way (See tutorial at the ExtraTrain web site). Palinsight is also a tool for searching palindromic patterns. To define the pattern the user selects the template sequence containing the desired pattern. The palindromic pattern is visually represented and the user can interactively select the positions in which strict conservation of sequence is required and the positions in which palindromy is the only constraint. The strategy of searching palindromy conservation independent of sequence conservation can reveal new patterns for binding sites in which the palindromicity is the crucial constraint. In any case Palinsight helps in the manual analysis of extragenic regions and it can be especially useful in the phase previous to the definition of a binding-site motif.

### Specific applications of ExtraTrain

**Figure 1:**
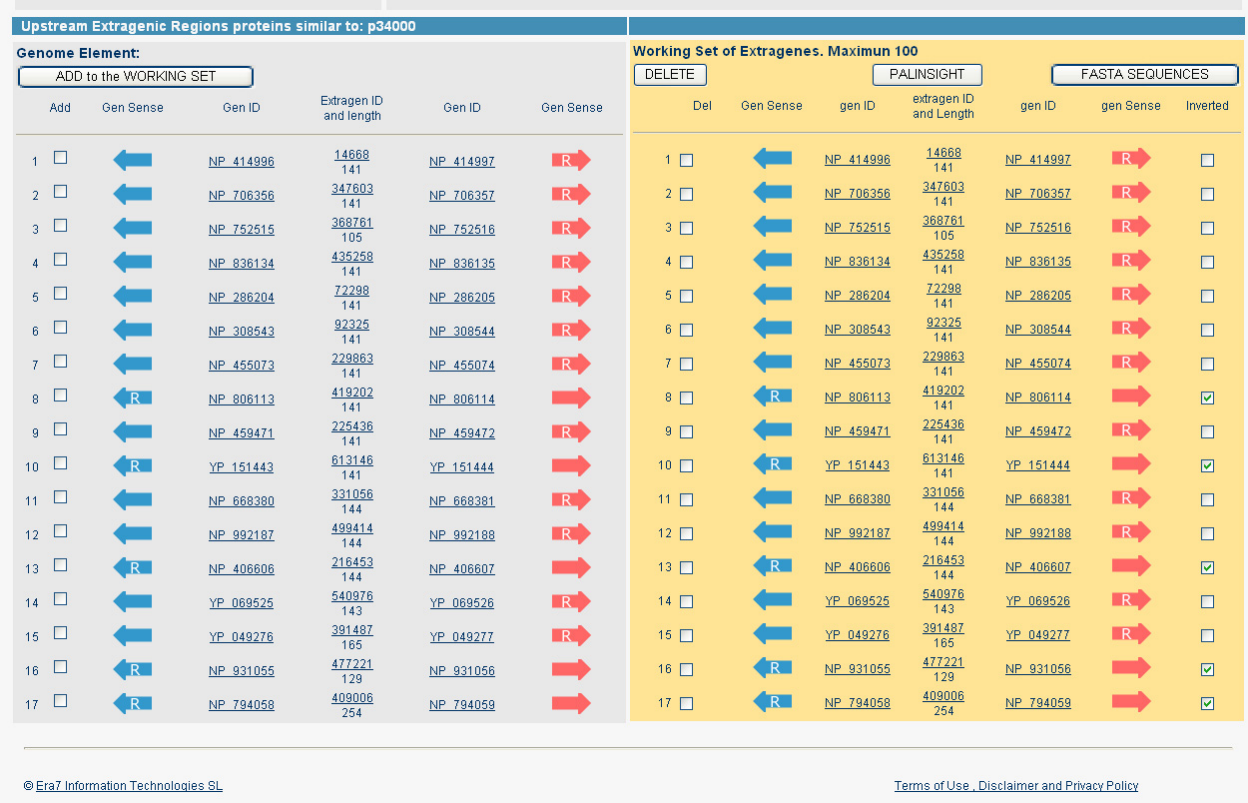
**Case study: constructing the working set**. The set of extragenic regions upstream genes encoding AcrR BLAST similar proteins has been incorporated to the "working set". For extragenic sequences 8, 10, 13, 16 and 17 the check-box for obtaining the complementary inverted sequence has been marked. Thus, the 17 upstream extragenic sequences are equally oriented with regard to the start points of the genes. Clicking on "FASTA SEQUENCES" button the user obtains the extragenic sequences in FASTA format. Clicking on "PALINSIGHT" button the user sends the sequences to Palinsight viewer.

**Figure 2:**
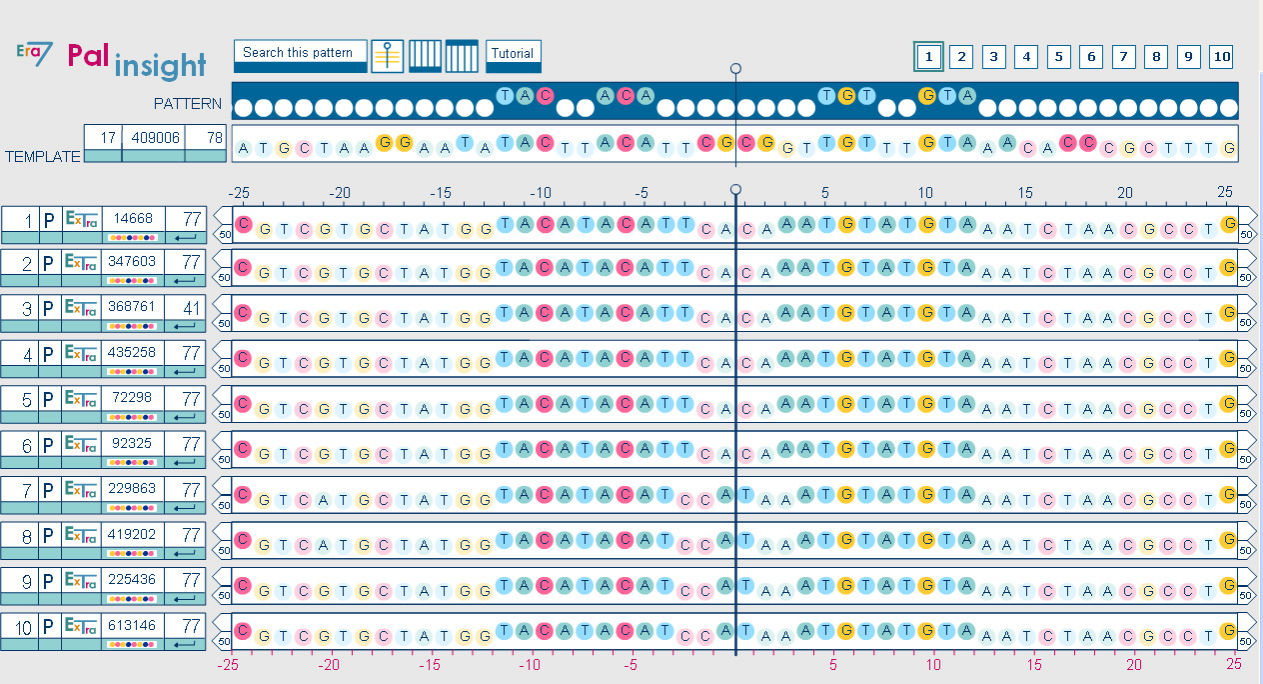
**Case study: Palinsight displaying the shared palindrome in sequences 1 to 10 of the working set**. Using Palinsight we have detected a shared palindrome in these extragenic sequences upstream genes encoding AcrR similar proteins. The same palindrome is conserved for all *Escherichia coli *and *Shigella flexneri *sequences (extragenic sequences 1–6). Another slightly different palindrome is conserved in *Salmonella enterica *and *Salmonella typhimurium *(extragenic sequences 7–10).

**Figure 3:**
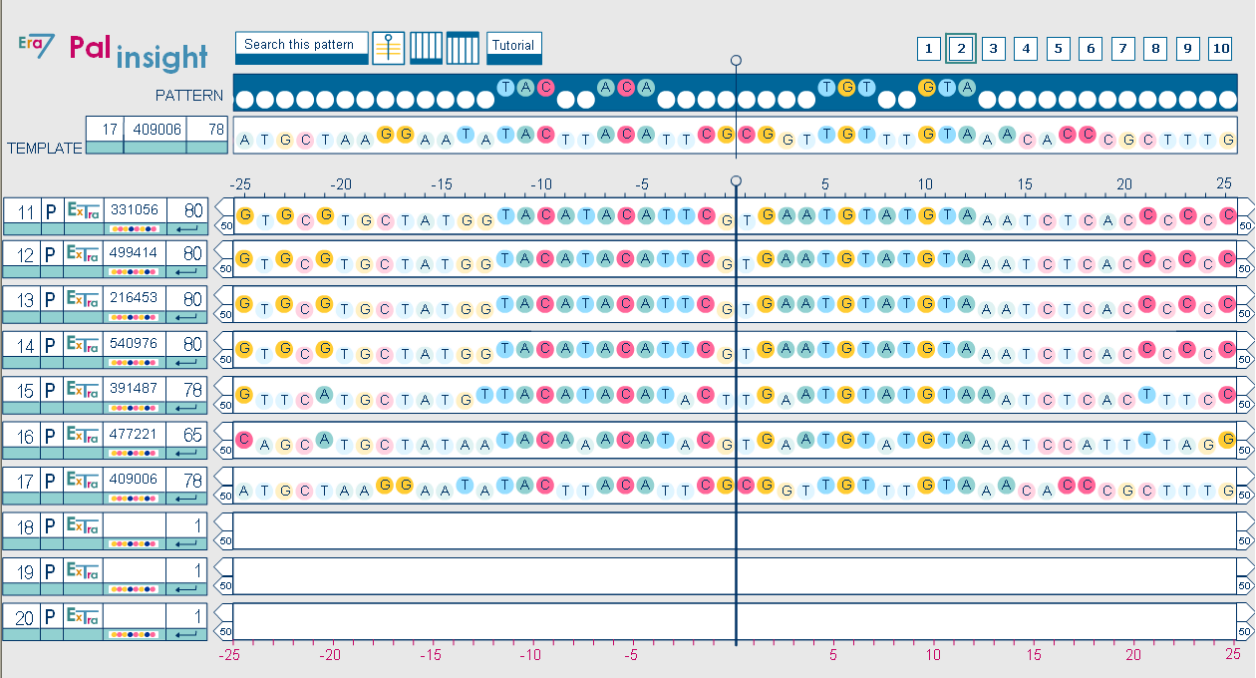
**Case study: Palinsight displaying the shared palindrome in sequences 11 to 17 of the working set**. Extragenic sequences 11–14 from *Yersinia *present another identical palindrome. Palindromes detected for *Erwinia carotovora*, *Photorhabdus luminescens *and *Pseudomonas syringae *present more differences but the 17 extragenic sequences conserve the palindromic motif TAC - -ACA- - - -|- - - -TGT - -GTA that appears at the top of the figures 2 and 3. The detected palindromes are candidates to be binding-sites for AcrR and AcrR similar proteins.

**Figure 4:**
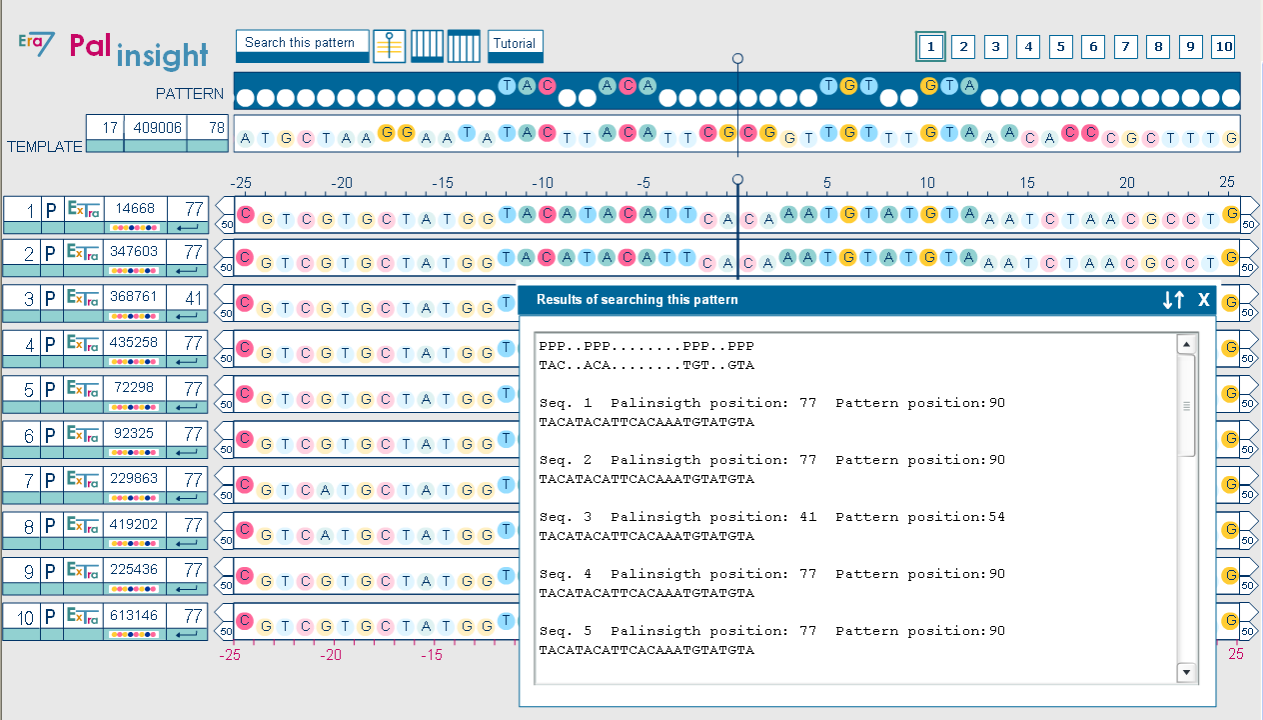
**Case study: Palinsight displaying the window of Results of searching the pattern**. When we searched for the pattern TAC - -ACA- - - -|- - - -TGT - -GTA clicking on the "Search this pattern" button we obtained the positions of this motif in the set of selected extragenic sequences. Table 3 is the copy of the complete content of this window.

ExtraTrain is oriented to facilitate the complex process of hypothesis driven experimentation and is especially designed for experimental scientists. In general, ExtraTrain manages sequences and information about extragenic regions and regulatory proteins of genomes of bacteria and archaea. Some research tasks are especially suited to be managed by ExtraTrain:

• Analysis of the extragenic regions corresponding to a set of differentially co-regulated genes. Gene expression data obtained from microarray experiments can be used as the raw data. Introducing either the RefSeq or the UniProt identifier, the user can obtain the corresponding extragenic regions and add them to the "working set". Then, these sequences can be sent to Palinsight to be analyzed. Thus, in an interactive step by step process, common motifs can be identified in the set of co-regulated genes.

• Searching for common features in the extragenic regions corresponding to a family of regulators. ExtraTrain offers the tools needed to study specific features of the binding sites corresponding to a family of regulatory proteins.

• Searching for repetitive extragenic palindromic (REP) sequences [17] in a genome element.

• Searching for terminators.

• Definition of binding sites for global regulators.

• Analysis of insertion sites of Insertion Sequence elements. Selecting the upstream and downstream extragenic regions of several copies of an Insertion sequence the user can analyze and compare their inverted repeats and the features of the insertion sites.

• Analysis of the extragenic regions corresponding to a set of BLAST similar transcriptional regulators. Bacterial transcriptional regulators usually autoregulate their own expression. Hence, it is probable to find similar signals in the DNA regions upstream a set of similar transcriptional regulatory proteins. ExtraTrain allows the visualization and comparison of these extragenic regions. In the figures 1, 2, 3, 4 we have represented a case study in which ExtraTrain is used to search for binding sites for regulatory proteins similar to AcrR of *Escherichia coli*. In the AcrR entry page, the user can automatically get the upstream extragenic regions corresponding to the set of AcrR BLAST similar proteins and then construct a "working set" with these sequences. In the "working set" page the user can observe the orientation of each gene and select each extragenic sequence in the appropriate orientation. Then, the correctly oriented sequences can be sent to Palinsight to be explored. Figures 2 and 3 display the similar palindromes detected at a similar distance of the gene start point, for the 17 AcrR similar sequences. These palindromes had been proposed as putative AcrR binding-sites for *E. coli*, *Salmonella typhi *and *Yersinia pestis *[18]. Using Palinsight we have found similar palindromes for *E. coli *K12, CFT073, O157:H7 EDL933 and O157:H7, *Shigella flexneri *2a 301 and 2457T, *Salmonella enterica *subsp. enterica serovar *Typhi *CT18, *Typhi *Ty2 and serovar *Paratyphi *A ATCC 9150, *Salmonella typhimurium *LT2, *Yersinia pestis *KIM, Medievalis 91001 and CO92, *Yersinia pseudotuberculosis *IP 32953, *Erwinia carotovora subsp. atroseptica *SCRI1043, *Photorhabdus luminescens subsp. laumondii *TTO1 and *Pseudomonas syringae pv. tomato *str. DC3000. (Tables 2, 3 and Figures 1, 2, 3, 4). The shared palindromic motif is indicated in the figures 2, 3, 4 and in Table 3.

### Future directions

ExtraTrain is a platform open to the contribution of knowledge by the scientific community. This collaborative system is our strategy to face the challenge of achieving a sustainable and maintainable knowledge database.

## Conclusion

ExtraTrain is a web platform to easily manage extragenic regions and transcriptional regulators in bacteria and archaea.

## Availability and requirements

ExtraTrain is freely available for research activities and non-commercial use at . The only requirement to use ExtraTrain is to have Macromedia Flash Player 7 or higher. The majority of web browsers have installed Flash Player but, in any case the user can download it at .

## Authors' contributions

EP has developed the web application, has contributed to the general design of the database and the web interface and has participated in the reviewing of the manuscript. PP-T has developed the Java programs to parse the Uniprot raw file and the NCBI genome files in order to extract the data and construct the database. MM has manually extracted and curated the references of the database. EP-T has collaborated in the management of references, Palinsight depuration, tutorial development and reviewing of the manuscript. JB has programmed the user management system. RT has contributed to the general design of the database and the user interface, has programmed Palinsight, has supervised the knowledge content of the database and has written the manuscript.

## Figures and Tables

**Table 1:** Families of Transcriptional regulatory proteins in bacteria and archaea.

Family	InterPro entry	ExtraTrain entries	Action	Structural motif	DBD position
AraC/XylS	IPR000005 HTHAraC	2485	Activator	HTH	C-terminal
ArsR	IPR001845 HTH_ArsR	982	Repressor	HTH	Central
AsnC	IPR000485 HTH_AsnC_lrp	803	Dual	HTH	N-terminal
Cold shock domain	IPR002059 Cold_shock	607	Activator	RNA- binding like	Variable
CRP-FNR	IPR001808 HTH_Crp	414	Activator/Dual	HTH	C-terminal
DeoR	IPR001034 HTH_DeoR	680	Repressor	HTH	N-terminal
GntR	IPR000524 HTH_GntR	1989	Repressor	HTH	N-terminal
IclR	IPR005471 HTH_IclR	538	Repressor	HTH	N-terminal
LacI	IPR000843 HTH_LacI	1079	Repressor	HTH	N-terminal
LuxR	IPR000792 HTH_LuxR	2117	Activator	HTH	C-terminal
LysR	IPR000847 HTH_LysR	3864	Dual	HTH	N-terminal
MarR	IPR000835 HTH_MarR	1316	Dual	HTH	Central
MerR	IPR000551 HTH_MerR	1112	Repressor	HTH	N-terminal
NtrC/Fis	IPR002197 HTH_Fis	3089	Activator	HTH	C-terminal
OmpR	IPR001867 Trans_reg_C	2253	Activator	winged helix	C-terminal
TetR	IPR001647 HTH_TetR	2718	Repressor	HTH	N-terminal

This table contains information about the 16 families of transcription factors included in ExtraTrain database. The first column contains the name of each family. The second column contains the identifier of the InterPro entry that defines each family. The third column contains the number of members of each family included in the database. The fourth column indicates if the members of this family usually are activators, repressors or have a dual action. The fifth column indicates the protein structural motif involved in the DNA interaction. The last column indicates the N or C-terminal position of the DNA-binding domain in the sequence of the regulatory protein.

**Table 2:** Extragenic regions upstream genes encoding AcrR similar proteins

	regulator ID	Gene Name	genome	Extra. region length
1	NP_414997	acrR	*Escherichia coli K12*	141
2	NP_706357	acrR	Shigella flexneri 2a str. 301	141
3	NP_752516	acrR	Escherichia coli CFT073	105
4	NP_836135	acrR	Shigella flexneri 2a str. 2457T	141
5	NP_286205	acrR	*Escherichia coli *O157:H7 EDL933	141
6	NP_308544	acrR	*Escherichia coli *O157:H7	141
7	NP_455074	acrR	*Salmonella enterica subsp. enterica serovar Typhi *str. CT18	141
8	NP_806113	acrR	*Salmonella enterica subsp. enterica serovar Typhi *Ty2	141
9	NP_459472	acrR	*Salmonella typhimur*ium LT2	141
10	YP_151443	acrR	*Salmonella enterica subsp. enterica serovar Paratyphi A *str. ATCC 9150	141
11	NP_668381	acrR	*Yersinia pestis *KIM	144
12	NP_992188	acrR2	*Yersinia pestis biovar Medievalis *str. 91001	144
13	NP_406606	acrR	*Yersinia pestis *CO92	144
14	YP_069526	acrR	*Yersinia pseudotuberculosis *IP 32953	143
15	YP_049277	acrR	*Erwinia carotovora subsp. atroseptica *SCRI1043	165
16	NP_ 931055	acrR	*Photorhabdus luminescens subsp. laumondii *TTO1	129
17	NP_794058	PSPTO4302	*Pseudomonas syringae pv. tomato str*.DC3000	254

This table contains some data extracted from the entries corresponding to the extragenic regions that participate in the case study.

**Table 3:** Results of searching the Palinsight pattern

PPP..PPP........PPP..PPP
TAC..ACA........TGT..GTA
Seq. 1 Palinsigth position: 77 Pattern position:90
TACATACATTCACAAATGTATGTA
Seq. 2 Palinsigth position: 77 Pattern position:90
TACATACATTCACAAATGTATGTA
Seq. 3 Palinsigth position: 41 Pattern position:54
TACATACATTCACAAATGTATGTA
Seq. 4 Palinsigth position: 77 Pattern position:90
TACATACATTCACAAATGTATGTA
Seq. 5 Palinsigth position: 77 Pattern position:90
TACATACATTCACAAATGTATGTA
Seq. 6 Palinsigth position: 77 Pattern position:90
TACATACATTCACAAATGTATGTA
Seq. 7 Palinsigth position: 77 Pattern position:90
TACATACATCCATAAATGTATGTA
Seq. 8 Palinsigth position: 77 Pattern position:90
TACATACATCCATAAATGTATGTA
Seq. 9 Palinsigth position: 77 Pattern position:90
TACATACATCCATAAATGTATGTA
Seq. 10 Palinsigth position: 77 Pattern position:90
TACATACATCCATAAATGTATGTA
Seq. 11 Palinsigth position: 80 Pattern position:93
TACATACATTCGTGAATGTATGTA
Seq. 12 Palinsigth position: 80 Pattern position:93
TACATACATTCGTGAATGTATGTA
Seq. 13 Palinsigth position: 80 Pattern position:93
TACATACATTCGTGAATGTATGTA
Seq. 14 Palinsigth position: 80 Pattern position:93
TACATACATTCGTGAATGTATGTA
Seq. 15 Palinsigth position: 78 Pattern position:91
TACATACATACTTGAATGTATGTA
Seq. 16 Palinsigth position: 65 Pattern position:78
TACAAACATACGTGAATGTATGTA
Seq. 17 Palinsigth position: 78 Pattern position:91
TACTTACATTCGCGGTTGTTTGTA
